# Relationships between social capital, patient empowerment, and self-management of patients undergoing hemodialysis: a cross-sectional study

**DOI:** 10.1186/s12882-022-02669-y

**Published:** 2022-02-21

**Authors:** Yongchao Hou, Li Li, Qian Zhou, Guohong Wang, Rongshan Li

**Affiliations:** 1 Emergency Department, ShanXi Provincial People’s Hospital, No. 29 Shuang Ta East Street, Taiyuan, 030000 ShanXi China; 2 Department of Nephrology, ShanXi Provincial People’s Hospital, No. 29 Shuang Ta East Street, Taiyuan, 030000 ShanXi China

**Keywords:** End-stage renal disease, Hemodialysis, Mediation effect, Patient empowerment, Self-management, Social capital

## Abstract

**Background:**

Hemodialysis is the most common treatment of end-stage renal disease. However, it is associated with a range of symptoms affecting patients’ daily activities and quality of life. Effective self-management has proven crucial for the alleviation of symptoms. According to Social Cognitive Theory, social capital and patient empowerment may be important variables for predicting self-management. To date, few studies have explored the mechanisms underlying these results. The study aimed to verify whether patient empowerment mediated the effect of social capital on the self-management of hemodialysis patients.

**Methods:**

The study was performed with 245 hemodialysis patients from January 2021 to April 2021 in Taiyuan, China. Demographic and clinical characteristics, social capital, patient empowerment, and self-management of patients undergoing hemodialysis were measured with a self-reported questionnaire. Descriptive statistics were used to summarize the participants’ demographic and clinical characteristics, and bootstrapping tests were used to verify whether patient empowerment mediated the association of social capital with self-management in patients undergoing hemodialysis.

**Results:**

Mediation analysis indicated that social capital and patient empowerment significantly predicted self-management. Patient empowerment partially mediated the relationship between social capital and self-management in hemodialysis patients.

**Conclusions:**

The results suggest that hemodialysis patients show relatively poor self-management and that patient empowerment mediates both social capital and self-management. Strategies to mobilize patients’ social networks and help them identify and utilize effective social resources may provide useful information regarding the implementation of optimal health management for their disease.

## Background

Chronic kidney disease, characterized by gradually declining kidney function [1], is increasingly becoming a global health issue. End-stage renal disease (ESRD) represents the advanced stage in which renal function progresses to a state of failure. Renal transplantation is the preferred treatment for ESRD [2]. However, this treatment is not always feasible due to limited numbers of donors, lack of appropriate medical facilities, or the ineligibility of recipients for transplantation [3–6]. Therefore, hemodialysis (HD) has become the most common treatment to prolong the survival of patients. According to statistics, approximately 90% and 86% of the dialysis population are undergoing HD in the USA and China, respectively [7, 8]. Although HD is lifesaving, patients on HD endure a range of symptoms, such as fatigue, pain, sleep disturbance, itching, anorexia, vomiting, nausea, constipation, anxiety, and depression [9–11], affecting their daily activities, well-being, and quality of life [11–14]. In order to alleviate these symptoms, HD patients are required to follow a unique and restrictive treatment regimen, including regular attendance at a dialysis unit for treatment, monitoring of diet and fluid intake, and medication restriction [15]. These lifestyle changes require a considerable degree of self-management [16].

Self-management is defined as the ability of a given patient to manage the illness, change lifestyle, and thereby live with a chronic illness [17]. The self-care compliance of hemodialysis patients has a direct influence on their health status and quality of life [18]. Self-management levels, especially during the interdialysis period, are strongly related to patient outcomes [19, 20], and also determine the treatment methods and ultrafiltration quantity during subsequent dialysis procedures [21]. However, self-management levels at present have been shown to be suboptimal in hemodialysis patients, particularly in low‐ and middle‐income countries such as China [15, 22]. A deeper understanding of factors associated with self-management is warranted [23]. Accordingly, this study was conducted to explore one potential mechanism of self-management, thereby providing useful knowledge for implementing targeted strategies to enhance self-management in patients with HD.

Social capital broadly refers to the relationships, social networks, support systems, and resources to which a given individual has access [24] and is composed of bonding social capital and bridging social capital. Bonding social capital refers to the emotional or substantive support‐based resources between strongly tied persons (e.g., family and closest friends), whereas bridging social capital concerns the information‐based benefits between weakly tied persons in heterogeneous networks (e.g., colleagues, acquaintances, and friends in general) [25, 26]. The importance of social capital as a determinant of health outcomes has been a topic of increasing research interest in recent years. From an interpersonal perspective, social capital can create a supportive environment that enables patients to take a more active role in their care management [27]. For example, studies have documented that social capital can facilitate improved self-management among patients with type 2 diabetes and HIV [28, 29]. Social capital was also positively correlated with the adherence to treatment or management plans in individuals with chronic diseases [30, 31]. In a qualitative study performed on hemodialysis patients, social capital was proposed to be conducive to their medication management [32]. However, few quantitative studies have reported the relationship between social capital and self-management in hemodialysis patients, especially in the context of Chinese culture. Thus, we hypothesized that social capital might exert a direct positive effect on self-management in patients undergoing HD in China.

A better understanding of how social capital impacts on self-management of HD patients is necessary. Social Cognitive Theory [33] indicates that individual behavior does not simply respond to, nor is solely influenced by the external environment. Instead, it is mediated to a large extent by cognition, and the behavior of an individual is the outcome of bidirectional interactions between personal, environmental, and behavioral factors. As a previous study suggested, self-concept, as the core of the self-cognition system, is affected by the external environment of parental rearing styles. Furthermore, it has subjective initiative, and together with external environment, affects middle school students’ learning burnout (behavioral outcomes) [34]. In the present study, patient empowerment was seen as a critical aspect of the self-cognition system, and we proposed that patient empowerment was likely to be a mediator of social capital (external environment) and self-management (behavioral outcomes). Patient empowerment in a healthcare context refers to the degree to which patients are able to act autonomously and think critically concerning their own treatment and care [35]. The concept of patient empowerment emphasizes the patient-centeredness of health care, stressing that the patient has the right to choose their treatment instead of simply complying with the offered treatments. In the empowerment process, healthcare providers offer accurate and comprehensible information and patients can access and screen multiple information to help them identify their strengths, thereby allowing them to determine and manage their disease [36]. Prior studies have confirmed that social capital is correlated with patient empowerment and that individuals with greater social capital are more likely to perceive themselves as empowered [27]. However, there appear to be no studies on the relationship between social capital and patient empowerment in HD patients. Therefore, we hypothesized that social capital was likely to be positively associated with patient empowerment in HD patients.

Additionally, patient empowerment has been shown to be associated with better health outcomes [37]. It has been suggested that patients with higher health literacy, empowerment predisposes them to better health management [38]. Empowerment is an important psychological factor and has been found to play a positive role in various health fields. A body of empirical literature on diabetes patients has suggested that empowerment can substantially alter their self-care behaviors [39–41]. Along the same lines, empowerment was found to actively impact on the self-management practices of asthma patients [42]. To sum up, there is evidence that patient empowerment is related to the self-management of several chronic diseases. We thus hypothesized that patient empowerment may be an important predictor of self-management in patients receiving HD.

Based on the above theory and literature, we conducted a survey of HD patients, with the aim of quantifying HD patients’ self-management and identifying the underlying factors contributing to self-management. We first hypothesized that there was an association between social capital and self-management. Moreover, we speculated that this relationship was mediated by patient empowerment. To our knowledge, the relationships among these variables have not been investigated in patients receiving HD in China.

## Methods

### Study design

This was a cross-sectional study, aiming to interpret the correlations between social capital, patient empowerment, and self-management, to identify the underlying path of social capital to self-management in patients receiving HD, and to verify whether patient empowerment acts as a mediator between social capital and self-management in patients receiving HD.

### Setting and participants

The study participants were recruited from two dialysis units at tertiary Level-A hospitals located in Taiyuan, China using a convenience sampling approach from January 2021 – April 2021. The sample size was predetermined on the basis of Kendall’s principle that the sample size should be 5–10 times the number of variables [43]. As this study included a total of 17 variables, including demographic/clinical variables, the estimated minimum sample size was 85. Allowing a 20% loss to follow-up, 258 patients were thus enrolled in the study. The inclusion criteria were patients who: (1) had undergone routine hemodialysis for at least three months; (2) were 18 years of age or older. The exclusion criteria were patients who: (1) exhibited hearing impairments; (2) were unable to communicate in Chinese; (3) suffered from psychological or cognitive disorders; (4) had physical limitations associated with their self-care; (5) were without arteriovenous fistulae, as the items of 5, 9, 15 in the self-management scale for hemodialysis patients are related to arteriovenous fistulae, such as item 5 “taking care of arteriovenous fistula”. Nine participants refused to cooperate during the investigation, three participants were unable to continue to attend the survey due to deterioration of illness, and one suffered from mild depression. Therefore, the final sample included 245 patients (participation rate = 94.96%).

### Procedure

The entire survey was conducted by three trained surveyors who only knew the items of the questionnaire instead of the purpose and process of the research. The surveyors firstly met with the potential participants and discussed the study goals, background, significance, and confidentiality. After signing an informed consent document, the participants filled in the self-reported questionnaire, specifically, questions on demographic and clinical characteristics, social capital, patient empowerment, and self-management according to their own situation. If patients were unable to accurately understand an item in the questionnaire, the surveyors provided explanations and instructions. The surveyors also assisted patients with difficulty with their hands in filling in the questionnaires. In both cases, the surveyors strictly followed the participants’ wishes without induced descriptions. Finally, they carefully evaluated each questionnaire to ensure that it had been fully completed. The Ethics Committee of Provincial People’s Hospital approved this study (Approval No. [2021]48), and all protocols were consistent with the 1964 Helsinki Declaration and its later amendments or comparable ethical standards.

### Study instrument

#### Personal characteristics

The patients’ demographic/clinical variables regarding age, sex, educational level, duration of dialysis, and comorbidities were collected.

#### Social capital

The social capital of HD patients was measured using the Chinese revised version of the Social Capital Scale, which was originally developed by Williams [44] and applied to Chinese patients in 2019 [27]. The scale was used to assess both online and offline social capital. The present study adopted offline settings of the scale to assess the social capital of HD patients. The Chinese revised version of the scale contains 15 items and 2 subscales: bonding social capital and bridging social capital. All items in the scale are rated on a 5-point scale from 1 (strongly disagree) to 5 (strongly agree). The overall scores are calculated by adding all individual scores together, with higher scores corresponding to more social capital. It has good psychological measurement characteristics [27]. In our study, the Cronbach’s α for this total scale was 0.897 and confirmatory factor analysis showed that the structure for the scale was stable.

#### Patient empowerment

In order to assess perceptions of patient empowerment in individuals receiving HD, we adopted the Chinese version of the Client Empowerment Scale [36], which was originally developed by Mikky [45]. The Chinese version of the scale contains 44 items and 6 dimensions: informed confidence, client-provider relationship, social advocacy, awareness, control, and client–client support. The items are scored on a 5-point scale from 1 (strongly disagree) to 5 (strongly agree), with the overall scores being the sum of the individual scores. Higher scores correspond to greater levels of empowerment. This scale has been widely used among patients with chronic diseases and has been proven to have good psychological properties [36]. In this study, the Cronbach’s α was 0.926.

#### Self-management

Self-management in the study subjects was assessed with the self-management scale for hemodialysis patients [46], adopting the Chinese version of this scale for the present analyses [15]. This scale is composed of 20 items and 4 subscales: partnership, problem-solving, self-care, and emotional management. The patients were instructed to respond to each item on a 4-point Likert scale ranging from 1 (never) to 4 (always), with the total score being used to assess the overall self-management capacity of a given patient. Higher scores indicated higher levels of self-management. The Chinese version of this scale has been demonstrated to exhibit good reliability and validity when used to assess Chinese HD patients [15]. The Cronbach’s α of the total scale in this study was 0.866.

#### Data analysis

The independent-samples t-test or the one-way ANOVA was used to analyze the distribution of self-management based on different demographic/clinical characteristics when the data were normally distributed and showed homogeneity of variance. The normality of the data distribution was determined using the Kolmogorov–Smirnov test, and normally distributed data were reported as means and standard deviations. The main variables in this study tested by Kolmogorov–Smirnov conformed to normality (social capital, *p* = 0.062; patient empowerment, *p* = 0.079; self-management, *p* = 0.200). Pearson correlation coefficients were then used to assess the associations between social capital, patient empowerment, and self-management. The mediating analysis was finally performed with PROCESS macro in SPSS [47]. The age and educational level which were associated with self-management of hemodialysis patients in the results of the previous studies were ranked as covariate variables in this study [16, 48]. Therefore, we put the age, educational level into the ‘covariates’ to control, self-management was the dependent variable, social capital was the independent variable and patient empowerment was the mediating variable when conducting the bootstrap process. The mediation effect was tested by the bootstrap resampling method, and 5000 bootstraps and 95% confidence intervals (CIs) were used to assess the mediation effect with PROCESS v3.3 (by Andrew F. Hayes) of SPSS 23.0. The 95% confidence intervals of the indirect effect did not contain zero and the mediating effect was statistically significant. Two-tailed p-values < 0.05 indicated statistical significance.

## Results

### Differences in self-management based on participant demographic/clinical characteristics

The associations between participant demographic/clinical characteristics and self-management scores were outlined in Table 1. As shown in Table 1, most participants were 60 years or older (37.1%). Most participants were men (60.8%), had received middle school education or less (40.0%), had received regular dialysis for 1–4 years (42.0%), and accompanied with comorbidities (84.5%). Considering the demographic and clinical characteristics, the self-management scores differed significantly according to age (*F* = 5.774, *p* < 0.001) and educational level (*F* = 10.008, *p* < 0.001). The overall trend was that younger and more highly educated participants had higher levels of self-management.

**Table 1:** Association between participant characteristics and self-management scores (*N* = 245)

Variable	*N(%)*	*M(SD)*	*F(t)*	*P*
Age (years)				
< 30	24 (9.8)	55.63 (11.23)	F = 5.774	< .001
30–39	49 (20.0)	58.80 (11.83)		
40–49	36 (14.7)	56.36 (10.62)		
50–59	45 (18.4)	51.11 (9.67)		
≥ 60	91 (37.1)	50.73 (10.86)		
Sex				
Male	149 (60.8)	53.87 (10.71)	t = .259	.796
Female	96 (39.2)	53.48 (12.15)		
Educational level				
Middle school or less diploma	98 (40.0)	50.19 (10.68)	F = 10.008	< .001
High school diploma or graduate equivalency diploma	69 (28.2)	54.45 (10.21)		
College school or higher diploma	78 (31.8)	57.50 (11.69)		
Duration of dialysis (years)				
< 1	101 (41.2)	54.47 (12.28)	F = .484	.694
1–4	103 (42.0)	53.65 (10.21)		
5–9	31 (12.7)	52.39 (11.18)		
≥ 10	10 (4.1)	51.00 (12.28)		
Comorbidities				
Yes	207 (84.5)	53.46 (11.24)	t =—.840	.402
No	38 (15.5)	55.13 (11.51)		

*M*, mean score *SD*, standard deviation. Comorbidities refer to those diseases that accompany with ESRD and that can reduce the survival rates and quality of life of patients [15, 16]

### Correlations between social capital, patient empowerment, and self-management

As shown in Table 2, positive correlations between social capital (*r* = 0.394), patient empowerment (*r* = 0.608) and self-management were observed, with an additional positive correlation seen between social capital and patient empowerment (*r* = 0.478).

**Table 2:** Distributions and correlations among the main study variables (*N* = 245)

Variable	*M* (*SD*)	1	2	3
1 Social capital	52.98 (11.04)	1		
2 Patient empowerment	158.63 (20.23)	.478 (< .01)	1	
3 Self-management	53.72 (11.28)	.394 (< .01)	.608 (< .01)	1

*M*, mean score *SD*, standard deviation

### Mediation effect of patient empowerment on the relationship between social capital and self-management

**Fig. 1:**
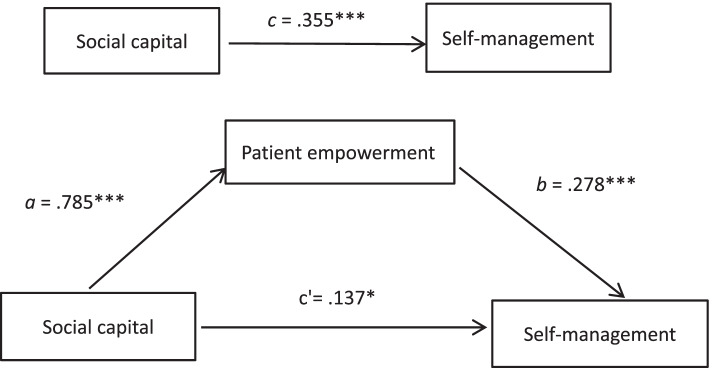
Mediation effect of patient empowerment between social capital and self-management. Path c indicates total effect of social capital on self-management, Path a * b indicates indirect effect of social capital on self-management via patient empowerment, Path c' indicates direct effect of social capital on self-management without patient empowerment. *p < .05, **p < .01,***p < .001

The mediation effect results were shown in Tables 3 and 4. The results showed that the total effect (path c) of social capital on self-management was 0.355 ( *p* < 0.001). The coefficients of path a (*B* = 0.785, *p* < 0.001, 95%CI [0.585, 0.985]) and path b (*B* = 0.278, p < 0.001, 95% CI [0.213, 0.343]) were significant, indicating that social capital was positively correlated with patient empowerment and patient empowerment was positively correlated with self-management. In addition, the indirect effect (a * b) between social capital and self-management via patient empowerment was 0.218 (95% CI [0.115, 0.335]). Therefore, the mediation effect accounted for 61.41% of the total effect of social capital and self-management. The direct effect of social capital on self-management was 0.137 (*p* < 0.05, 95% CI [0.023, 0.252]), indicating that patient empowerment partially mediated the association between social capital and self-management. The final mediation model was shown in Fig. 1.

**Table 3:** The unstandardized regression coefficients (*B*) with Standard Errors (*SE*) and 95% confidence intervals (*CI*) estimating patient empowerment and self-management

	Patient empowerment			Self-management		
	*B*	*SE*	*95%CI*	*B*	*SE*	*95%CI*
Constant	133.439	7.006	[119.639, 147.239]	7.452	5.691	[-3.759,18.664]
Age	-2.098	0.806	[-3.686,—0.511]	-0.968	0.420	[-1.794, -0.141]
Educational level	-4.315	1.373	[-7.020, -1.610]	-0.785	0.719	[-2.202, 0.632]
Social capital	0.785	0.102	[0.585, 0.985]	0.137	0.058	[0.023, 0.252]
Patient empowerment				0.278	0.033	[0.213, 0.343]
*R*^*2*^	0.294			0.403		
*F*	33.381			40.545		
*p*	< .001			< .001		

Effects are significant when the upper and lower bound of the 95% confidence intervals (CI) does not contain zero

**Table 4:** Summary of effects of mediation

Mediation	Effect (*B*)	Boot *SE*	Boot LLCI	Boot ULCI	Relative effect (%)
Path ab	.218	.057	.115	.335	61.41%
Path c′	.137	.058	.019	.023	38.59%
Path c	.355	.059	.239	.472	

Note: Path ab, indirect effect of social capital on self-management; Path c′, direct effect of social capital on self-management; Path c, total effect of social capital on self-management; Effect(*B*), unstandardized coefficient *BootSE*, standard error estimated using bootstrapping method *BootLLCI*, lower level of confidence interval estimated using bootstrapping method *BootULCI*, upper level of confidence interval estimated using bootstrapping method; Relative effect refers to the indirect effect accounts for 61.41% of the total effect, and the direct effect accounts for 38.59% of the total effect, respectively

## Discussion

Previous studies pointed out that self-management in HD patients was suboptimal, indicating a need for improvement [15, 22]. One of the important goals in HD treatment is the self-management of routine activities, and good self-management results improve both the individual’s well-being and health [49]. Besides, according to Social Cognitive Theory, social capital and patient empowerment were probably regarded as the critical factors affecting self-management in HD patients. Addressing these issues has been an essential part of patient care. To our knowledge, the associations between social capital, patient empowerment, and self-management have not been elucidated in a mediation model. The present study confirmed our earlier hypothesis, that is, social capital and patient empowerment were positively related to self-management in HD patients, and patient empowerment was found to play a mediating role between social capital and self-management, which provided an empirical reference for promoting the self-management of HD patients based on Social Cognitive Theory.

We found that the participants with greater social capital had higher levels of self-management, which mirrors the research conducted by Koetsenruijter et al. [50] suggesting that self-care was associated with the patient’s social organization, community, and information and emotional network. In addition, social capital has been found to be beneficial for the maintenance of a healthy lifestyle in patients with coronary heart disease [51]. In terms of cultural differences, family and kinship are highly valued in China compared with western countries, and patients suffering from poor health and economic hardship are relatively vulnerable and prefer to obtain support from their families. Under these conditions, the mobilization of the patient’s social networks and obtaining the material and spiritual support is quite essential and even meaningful. Nevertheless, it is noteworthy that these studies have adopted different social capital instruments to validate the relationship between social capital and self-management in different cultural contexts.

Our results showed a significant correlation between patient empowerment and social capital. Social capital affords individuals with chronic health issues more emotional and informational support, thereby improving their feelings of empowerment [52]. In addition, our study indicated that patient empowerment was positively associated with self-management. This is consistent with a previous study [42]. Patients who rate themselves positively are more capable of self-management. In contrast, individuals in some cases possess sufficient resources to move to a more healthy state [53], but could not effectively utilize these resources such that their health condition is perceived to be unmanageable. Healthcare professionals can thus help guide patients in a better way to access support from patient organizations and their families [54], thus leading to a better self-management behaviour.

As described earlier, patient empowerment partially mediated the association of social capital with self-management. The effect of social capital on self-management can be partly explained by patient empowerment. As we know, social capital can provide a stable social environment for accessing emotional support and health information. Bonding social capital including a closer connection with family members, relatives, and friends can help HD patients regain confidence during their illness, while bridging social capital indicating a larger social network provided by physicians, health organizations, and patients with the same disease can help them access useful information for their health. The specific forms of social capital serve as a supportive environment to empower patients by providing useful advice and tangible assistance that are conducive to the patients’ self-management skills and confidence, promoting improvement in the patients’ self-management behaviors [27].

Despite these encouraging results, there are some limitations to the study that need to be noted. First and foremost, a convenience sample of eligible participants was conducted, which may have led to a risk of bias and poor representation of the total population, thus limiting generalizability to other populations. Moreover, we only discussed the total scores of the three main variables and their relationships, and the relationships between the dimensions of each scale were not considered. Therefore, future studies should analyze the dimensions of each scale to make the results more comprehensive. Furthermore, the study only regarded demographic and clinical variables as control variables, other psychosocial variables that may also influence self-management. It is suggested to include as many control variables affecting self-management as possible to make the results more accurate. Finally, all data were collected through self-reports and were thus susceptible to bias, and subjective and objective evaluation criteria are considered more beneficial.

## Conclusions

This study extends our current knowledge of the self-management of HD patients by elucidating its underlying mechanisms based on Social Cognitive Theory. The findings suggest that strengthening patient empowerment is beneficial for improving self-management in relation to the social environments of HD patients. In addition, it is recommended that both social relations and decision empowerment should be taken into account in the treatment regimens of HD patients.

## Data Availability

The datasets used in the study are available from the corresponding author on reasonable request.

## Abbreviations

ESRD: End-stage renal disease
HD: Hemodialysis
M: Mean score
SD: Standard deviation
SE: Standard error
LLCI: Lower limit of 95% confidence interval
ULCI: Upper limit of 95% confidence interval
